# Rationale and description of Tied by Tiredness: A blended care intervention for fatigue after acquired brain injury

**DOI:** 10.1177/02692155251407318

**Published:** 2025-12-16

**Authors:** Ela Lazeron-Savu, Tom Smejka, Bert Lenaert, Jeanette Dijkstra, Claire Wolfs, Vera Schepers, Rudolf Ponds, Caroline van Heugten

**Affiliations:** 1 School for Mental Health and Neuroscience, Faculty of Health, Medicine and Life Sciences, 82246 Maastricht University, Maastricht, The Netherlands; 2 Department of Medical Psychology, Catharina Hospital, Eindhoven, The Netherlands; 3 Section Teaching and Innovation of Learning, Faculty of Psychology and Neuroscience, 5211 Maastricht University, Maastricht, The Netherlands; 4 Department of Health Promotion, Faculty of Health, Medicine and Life Sciences, 82246 Maastricht University, Maastricht, The Netherlands; 5 Department of Neuropsychology and Psychopharmacology, Faculty of Psychology and Neuroscience, 82246 Maastricht University, Maastricht, The Netherlands; 6 Department of Medical Psychology, Maastricht University Medical Center, Maastricht, The Netherlands; 7 Limburg Brain Injury Center, Maastricht, The Netherlands; 8 Department of Rehabilitation, Physical Therapy Science and Sports, 8124 University Medical Center Utrecht, Utrecht, The Netherlands; 9 Departement of Medical Psychology, Amsterdam University Medical Center, Amsterdam, The Netherlands

**Keywords:** Fatigue, brain injury, stroke, experience sampling methodology, rehabilitation

## Abstract

**Aim:**

The development of a new intervention designed to reduce persistent fatigue following acquired brain injury through personalised support.

**Rationale:**

Fatigue is a common and long-lasting consequence after brain injury. Evidence indicates that tailored, multimodal interventions targeting individual experiences are more effective than standardised approaches.

**Materials and procedures:**

The intervention combines real-time data collection using the Experience Sampling Method via a dedicated mHealth app with a workbook containing practical instructions and an online secure feedback environment. Patients complete eight short daily assessments for three consecutive days each week, collecting detailed information on fatigue and contextual factors such as mood, physical activities and social situations.

**Providers, setting, and delivery:**

Tied by Tiredness is delivered by psychologists or occupational therapists in rehabilitation or outpatient settings to adults with acquired brain injuries aged 18 and over. In face-to-face therapy sessions, patient-collected data are used to tailor personalised feedback and advice on strategies, emphasising collaborative decision-making and active engagement.

**Dose and personalisation:**

Patients attend six weekly 1-h sessions. Intervention strategies are adjusted to individual needs and goals throughout the programme, based on ongoing assessment data.

**Unique features:**

Continuous personalisation and the integration of real-time data into therapy sessions distinguish this intervention.

**Purpose and implications:**

The programme aims to provide insight into the personal and environmental factors that contribute to a person's fatigue, enabling patients to implement cognitive and behavioural strategies for effective daily fatigue management. Tied by Tiredness represents a novel, practical approach to supporting self-management after brain injury.

**Trial registration:**

Overview of Medical Research in the Netherlands (OMON), ID: NL-OMON21265

## Clinical message

- The Tied by Tiredness intervention provides individualised, blended-care support to help adults manage persistent fatigue following acquired brain injury.
- Real-time, app-based data on fatigue and its contributing factors is integrated into personalised feedback and advice on strategies to manage fatigue in daily life.

## Introduction

Fatigue ranks among the most prevalent consequences of brain injury and has been associated with lower quality of life and poorer neurological recovery.^1,2^ A recent systematic review of interventions for fatigue after traumatic brain injury concluded that evidence-based multifactorial and multimodal treatment approaches are lacking, but should be preferred over unifactorial approaches which are unlikely to be effective for everyone given the complex, multifactorial nature of fatigue.^3^ For this, we developed the Tied by Tiredness intervention as an answer to the urgent need for effective evidence-based, personalised, and multifactorial treatments for fatigue after brain injury.

The name Tied by Tiredness refers to the feeling reported by many patients of feeling stuck in fatigue, and to how fatigue restricts their daily functioning. The name also reflects how the flow of daily life may be dictated by fatigue, which is what this intervention tries to change by giving control back to the individual.

The Tied by Tiredness intervention was tested in a pilot study.^4^ Ten individuals (six men and four women) participated in this study. After the pilot study, several refinements were made to the intervention. The m-Path app replaced the earlier PsyMate app due to persistent technical problems such as participants not receiving notifications, and the number of daily notifications was increased from eight to 10 to obtain more detailed information about daily fatigue patterns and compensate for missed signals. Additionally, some assessment items were revised to resolve ambiguities, with the finalised set included in the Supplemental Material. Feedback is now provided exclusively through face-to-face sessions, since email-based feedback proved to be both less efficient for therapists and less personal for participants. Furthermore, the treatment protocol was expanded with more detailed information regarding post-injury fatigue and cognitive-behavioural techniques, while allowing some content to be tailored depending on case relevance.

A comprehensive description of the definite version of the intervention is missing. Therefore, the aim of the current paper is to outline the theoretical background and content of *Tied by Tiredness*, using a detailed case example. The article is structured according to the TiDiER framework as presented in Table 1.

**Table 1.** A summary of the tied by tiredness intervention in accordance with the TIDieR framework.

TIDieR component	Description	Application in this paper
*Brief* *n* *ame*	Name or title of the intervention	Tied by Tiredness, a six-week blended-care programme
*Why (* *r* *ationale)*	Rationale, theory, or goal of the intervention	Addressing persistent fatigue after brain injury with personalised support
*What (* *m* *aterials)*	Physical or informational materials used	mHealth app, workbook, online feedback platform
*What (* *p* *rocedures)*	Procedures and activities	Experience Sampling Method, weekly face-to-face feedback sessions
*Who* *p* *rovided*	Expertise, background, and training of providers	Psychologists and occupational therapists in rehab/outpatient settings
*How *	Modes of delivery	Combination of app-based sampling and personalised therapy sessions
*Where*	Location where intervention is delivered	Rehabilitation and outpatient environments
*When and how much*	Timing, duration, intensity	Six weeks, 10 daily assessments on three days per week, weekly sessions
*Tailoring*	Adaptations made to suit individual requirements	Data-driven personalised feedback and strategies based on patient input
*Modifications*	Changes made during intervention	Adjusted notification timing; use of face-to-face feedback
*How* w*ell (fidelity)*	Assessment of adherence and fidelity	Continuous monitoring via app data and session reports

This intervention is the subject of an ongoing trial (NL-OMON21265).^4^

## Why: Rationale

Fatigue after brain injury is a complex symptom determined by neurobiological, psychological, and social factors and their interactions.^5^ For instance, premorbid factors such as demographic variables and pre-injury fatigue,^6^ psychological factors such as depressive mood^7,8^ and social factors such as social support^9^ have all been associated with fatigue. This suggests that treatment should acknowledge the many faces of fatigue and its related factors, as these may influence the course of fatigue and may determine which treatment approach is to be followed for whom.^10^

The Experience Sampling Method offers a promising way to personalise treatment by capturing individual differences in fatigue.^11,12^ Using an *mHealth* app, participants complete brief questionnaires several times per day about their symptoms, mood, and activities. This method allows gaining insight into diurnal variability in fatigue and into the factors related to this variability, which may differ in nature and often vary between individuals. These factors may subsequently become personalised treatment targets.^13^ Therefore, this intervention addresses the clinical need for personalised, multimodal treatment strategies that reflect the complex, multifactorial nature of post-brain injury fatigue.

Tied by Tiredness is a six-week blended care intervention combining experience sampling method data collection with weekly personalised face-to-face feedback sessions. The goal of the intervention is to reduce fatigue in daily life by, on the one hand, offering the patient (and the therapist) detailed insight into the unique mix of factors associated with an individual's fatigue and, on the other hand, encouraging individuals to adopt cognitive and behavioural strategies to reduce fatigue in daily life.

## What: Materials and procedures

### Feasibility and protocol development

The feasibility and usability of the experience sampling method after brain injury has been demonstrated in multiple studies.^14–18^ To date, no experience sampling method intervention has been developed to treat fatigue after brain injury. In other clinical fields, experience sampling methods have been successfully used as a therapeutic tool over the past decade, for example in the treatment of depression,^19^ or as a support tool for caregivers of people with dementia.^20^ This is often combined with weekly consultations, in which feedback is given based on an individual's data by a researcher or therapist. In their randomised controlled trial, Kramer et al. (2014) examined whether a six-week intervention combining experience sampling method data collection with weekly personalised feedback was feasible and effective in individuals receiving pharmacological treatment for depression. They found that weekly experience sampling method data collection combined with experience sampling method derived feedback was effective in reducing depressive symptoms relative to control groups. The basis of our Tied by Tiredness intervention treatment protocol for fatigue after acquired brain injury originated from the treatment protocol used by Kramer et al. (2014). This protocol was customised for the specific context of fatigue after brain injury by modifying and adding fatigue-specific items in the experience sampling questionnaire and tailoring the feedback content. Additionally, the themes were adapted to reflect the context of post-brain injury fatigue. These adjustments were made by the research team in collaboration with healthcare professionals.

### Materials and content

Before the start of the intervention, the healthcare professionals receive a workbook. The workbook contains information about the intervention and feedback sessions, the mHealth application ‘m-Path’, and the online web environment of m-Path that allows the data of each patient to be presented in a personalised and visually attractive way. Further, the workbook contains psycho-education about fatigue after brain injury and some more in-depth explanation of cognitive behavioural principles that can be applied to fatigue such as identifying unhelpful thoughts. The personalised feedback sessions are discussed in detail in week-by-week chapters, with step-by-step explanations of what aspects of fatigue should or can be discussed and what aspects of these should be highlighted and visually presented within m-Path. The workbook will be made available on the website of the Limburg Brain Injury Centre when the currently ongoing trial has been completed (https://www.hersenletsellimburg.nl/en). More information on m-Path, a platform based on the experience sampling method to facilitate repeated mobile assessment and intervention in everyday life, can be found at https://m-path.io/landing/.

### Procedures

The procedure and characteristics of the intervention are summarised in Table 2. There are six weekly feedback sessions in total, with each session having a specific theme. Each session lasts up to 1 h, with the therapist spending the first 15 min reviewing the patient's collected data online in order to identify any meaningful patterns. The remaining 45 min are dedicated to direct patient interaction. A case example is further elaborated in the Supplemental Material.

**Table 2.** Procedure and characteristics of the tied by tiredness intervention.

Intervention	Component	Content
Week 1	Fatigue during the day (Session 1)	• Getting to know the patient (and healthcare professional)
		• Introduction to treatment, explaining the method and worksheets
		• Discussing information about fatigue after brain injury and limited exercise capacity
		• Gaining personalised insight into the course of fatigue during the day and the measurement period
Week 2	Fatigue during the day in relation to mood and compared with the first week (Session 2)	**•** Gaining insight into the course of fatigue over the measurement period and in comparison, with the previous period
		• Gaining insight into the course of fatigue in relation to mood (link between fatigue and certain mood states and emotions; link between unhelpful thoughts about fatigue and mood)
		• Discussing optional modules such as ‘Dealing with unhelpful thoughts’ (if patient encounters such thoughts)
Week 3	Fatigue during the day in relation to physical exertion and compared with previous weeks (Session 3)	• Gaining insight into the course of fatigue over the measurement period and in comparison, with previous periods
		• Gaining insight into the course of fatigue in relation to physical exertion
		Identifying and discussing unhelpful thoughts about exercise – Optional
		• Discuss the importance of relaxation – Optional
Week 4	Fatigue during the day in relation to mental effort and compared with previous weeks (Session 4)	Goals:
		• Gaining insight into the course of fatigue over the measurement period and in comparison, with previous periods
		• Gaining insight into the course of fatigue in relation to mental effort
		• Identifying and discussing unhelpful thoughts about exercise – Optional
Week 5	Fatigue during the day in relation to activities and compared with previous weeks (Session 5)	• Gaining insight into the course of fatigue over the measurement period and in comparison, with previous periods
		• Gaining insight into the course of fatigue in relation to different activities
		• Identifying and discussing unhelpful thoughts about engaging in certain activities – Optional
Week 6	Fatigue during the day in relation to social contacts and location and compared with previous weeks (Session 6)	**•** Gaining insight into the course of fatigue over the measurement period and in comparison, with previous periods
		• Gaining insight into the course of fatigue in relation to social contacts
		• Discussing dealing with the (social) environment
		• Detecting and discussing unhelpful thoughts about dealing with others – Optional

*Week, session 1* – the theme of the first week's session is fatigue during the day (see case introduction box). This session provides an introduction to the intervention and its purpose of providing better insight in fatigue patterns and under which circumstances fatigue occurs. It is also explained that these insights will then be used as a tool to reduce or gain more control over fatigue by encouraging patients to adopt personalised cognitive or behavioural strategies in daily life. The graphical representation of a person's experience sampling data concerning general fatigue, physical fatigue and mental fatigue is also illustrated in this in this session.

At a glance it is possible to see how fatigue progresses within a day and over days and weeks, and at which moments fatigue is at its highest or lowest. Each week, insights the participants have gained are reflected upon by looking at graphic information which is subsequently discussed. In addition, cognitive and behavioural strategies that may reduce fatigue are discussed and participants are encouraged to try these out in the upcoming week.

*Week, session 2 –* The theme of the second session is fatigue in relation to mood. The graphical timeline of the answers to the following experience sampling items are discussed: ‘I feel tired’, ‘I am depressed’. ‘I feel anxious’, ‘I am irritable’, ‘I feel powerless’, ‘I am brooding’ and ‘I am stressed’. By selecting two or more of these items simultaneously, e.g. ‘I feel tired’ and ‘I am depressed’, the m-Path interface allows graphically visualising potential associations between these variables in a highly intuitive way. Moreover, provided that there are sufficient data points, m-Path will also calculate person-specific correlation coefficients and *p*-values between the selected variables.

This session also includes information from cognitive behavioural therapy about identifying dysfunctional thoughts and advising on how to deal with them. The information is optional and can be used if there are mood complaints and/or when unhelpful thoughts about fatigue are detected*.* The cognitive behavioural information and advice is bundled in a separate document, which can be handed over to the participant.

Further, the m-Path interface allows zooming in on specific days or looking at more general fatigue patterns over multiple weeks in the course of fatigue, both for general fatigue and for physical and mental fatigue. The course of fatigue over the past weeks is a theme that recurs every week during the intervention from Week 2 onwards.

*Week, session 3 –* In the third week's session, fatigue in relation to physical effort and exertion is discussed. Here, the graphical timeline representation of previously mentioned questions about fatigue is discussed, as well as the question ‘Since the last notification, I have engaged in physical effort’. By selecting these questions, it can be seen whether there is a positive, negative, or no meaningful relationship between experienced fatigue and the level of physical effort. Furthermore, psycho-education is given about what relaxation is and the importance of alternating between strenuous and relaxing activities is discussed. Attention is also given to dealing with unhelpful thoughts – if present – about physical effort and relaxation. Possible exercises and relaxation techniques are discussed, such as body scan or breathing exercises. The relaxation information is optional, depending on the participant's needs and the therapist's assessment.

*Week, session 4 –* The fourth week's session theme is fatigue in relation to mental effort and exertion. Similar to Week 3, the relationship between fatigue and mental effort is discussed. In this week, if necessary, extra attention is paid to the importance of relaxation, the right amount of effort and taking breaks. If present, attention is given to dysfunctional thoughts about mental effort or relaxation. As in previous weeks, the course of different types of fatigue during the intervention is examined, and cognitive and behavioural strategies are again evaluated and discussed for the upcoming week.

*Week, session 5 –* In Week 5's session, fatigue is discussed in relation to daily activities. By selecting the question ‘What I am doing?’, m-Path displays the activities that have been filled in by a person as well as the relative time spent doing these activities within a pie chart. This way, it may become clearer, for instance, whether a person takes too many or too few breaks or spends most of his time doing a certain activity (e.g. watching TV). This question about daily activities can be answered using preset multiple-choice options as well as by using the option to fill in other, non-listed answers. The m-Path interface also allows visualising the different types of fatigue during each of the reported activities, allowing personalised insight in which activities are associated with the highest or lowest levels of fatigue. m-Path also provides the option of creating a word cloud graph of all entered activities. This is an intuitive way of depicting which activities are done very often, and which are not. The choice to discuss daily activities in Week 5 was deliberate in that it guarantees sufficient data from previous weeks to get a reliable picture of the relative share of different types of activities a person engages in, as well as their relation to fatigue.

*Week, session 6 –* The last week of the intervention focuses on social context and environments. In this week the experience sampling items ‘Where am I?’, ‘Who am I with?’, ‘I like being alone’, ‘I like being in this company’, ‘I would rather be in the company of others’ and ‘I feel comfortable’ are discussed. As in previous weeks, the items of general, physical and mental fatigue can be selected and visually represented in relation to one or more of the items about social context. In this way, it can be seen whether the location (where someone is) and/or company (who someone is with) is related to a person's fatigue experience. The frequency graph can also be used for questions regarding location and company.

This week also provides information on how to deal with and plan social contacts, given the influence of fatigue on social relationships and vice versa. It is advised to maintain social contacts and regularly do things with others, as this may be fulfilling and may give energy to a person. It is also indicated that asking for support and indicating one's boundaries to others is important. Furthermore, the fact that misunderstandings from the social environment regarding fatigue can arise is also discussed and advice is given on how to deal with this. An explanation is also given about what it means to be assertive, sub-assertive or aggressive (i.e. going beyond the boundaries of others). This information is optional and can be discussed depending on the needs and request for assistance of a person and the assessment of the healthcare professionals.

This week, extra attention is paid to the timeline graph of general, physical and mental fatigue over the past weeks. The insights gained over the past weeks are discussed and the effects of adopted strategies are evaluated. Finally, the entire intervention is also evaluated and the treatment is concluded.

## Who provided

The intervention can be provided by healthcare professionals (occupational therapists or psychologists) who have knowledge and experience (i.e. they work in a setting where people with brain injury are treated) with brain injury patients with fatigue complaints. This intervention requires specific competencies in cognitive-behavioural techniques, motivational interviewing, and activity analysis – core skills within the professional scope of psychologists and occupational therapists. Psychologists bring expertise in behavioural change strategies and psychological assessment, while occupational therapists contribute specialised knowledge in activity adaptation and environmental modification.

The healthcare professionals who agreed to provide the Tied by Tiredness intervention have received a training. This training covered the intervention protocol in detail and provided a comprehensive overview of how to use the m-Path interface. The training took about 1 h and was mainly given online by the researcher. In the event that there were questions or uncertainties from the healthcare professionals about the m-Path interface, an extra meeting was scheduled to address these.

## How provided

Tied by Tiredness is a blended-care intervention, consisting of individual data collection using the m-Path app based on the experience sampling method, combined with weekly face-to-face feedback sessions with the therapist.

## Where provided

The intervention is developed for individuals who experience fatigue following acquired brain injury such as traumatic brain injury or stroke and who are seeking treatment for fatigue. The Tied by Tiredness intervention can be provided to persons aged 18 years or older who are treated by psychologists or occupational therapists in rehabilitation settings or occupational therapy practices. Patients with an objectified form of brain injury (stroke or traumatic brain injury) and a score of four or higher on the Fatigue Severity Scale can be considered for treatment.

The treatment as usual group for fatigue will be given by occupational therapists and consists of psycho-education about fatigue after ABI and elements of ‘Niet-Rennen-Maar-Plannen’ (‘Don’t run, but plan’, in English).^21^ However, in practice, substantial differences in treatment exist between participants and across locations. Importantly, the duration of TAU will be recorded in detail for all participants.

## When and how much

The intervention spans a total of six weeks, with each week following a consistent routine composed of two main components: (a) the patient collects experience sampling data using the m-Path app, and (b) discusses the collected data with the therapist during a face-to-face session.

Using the m-Path app, patients engage in experience sampling method data collection for three consecutive days each week, either – and based on personal preference – Thursday, Friday, and Saturday; or Sunday, Monday, and Tuesday (to ensure both weekdays and a weekend day are represented in the measurements). During these three days, the app sends 10 notifications per day, prompting patients to respond to a series of statements, such as ‘I feel tired’ or ‘Since the last beep, I have engaged in mental effort’.

In the introductory session, patients are instructed to always respond to experience sampling items based on how they felt just before receiving the notification, rather than how they felt earlier in the day. The notifications are sent at random times between 8 a.m. and 10:30 p.m., and patients are advised not to alter their daily routines to accommodate the study. The start and end time for the daily notifications window can be adjusted according to the patient's usual bedtime. It is also explained that it is perfectly normal to miss some of the notifications, while simultaneously motivating patients to respond to as many notifications as possible in order to obtain rich and reliable data.

## Tailoring

Tied by Tiredness aims to alleviate fatigue by providing both patients and therapists with detailed insights into each individual's unique combination of factors that may contribute to fatigue in daily life. Additionally, it offers a broad set of cognitive and behavioural strategies to reduce fatigue and enhance daily functioning, which can be used or not used depending on the identified factors. This intervention stands out because it is inherently tailored and specifically designed for personalised intervention in the daily lives of patients. For instance, used strategies may be aimed at improving physical fitness, increasing participation in certain (e.g. social) activities, improving daily planning, enhancing sleep hygiene, or addressing unhelpful thoughts related to fatigue through cognitive-behavioural principles. It offers a flexible toolkit that adapts to each patient's unique situation, grounded in the latest scientific insights and recommendations for effective interventions. It should also be stressed that this intervention is a collaborative effort between patient and therapist. Because patients collect their own data and are encouraged to reflect on their weekly progress, they are put in charge of their own rehabilitation progress. In that sense, the therapist takes the role of a coach who motivates patients to take back control over their fatigue by implementing and evaluating new strategies to reduce or cope with fatigue on a weekly basis.

The intervention is characterised as multimodal because it encompasses multiple therapeutic components targeting interrelated mechanisms of change. The intervention facilitates the development of individualised insight into the temporal course of fatigue and its associations with various psychosocial determinants. Participants are guided to identify patterns linking fatigue with mood, emotional states, and maladaptive cognitions, as well as with levels of mental effort and physical exertion. They are further encouraged to explore and critically discuss unhelpful thoughts and behaviours related to fatigue, exertion, relaxation, and social engagement. In addition, participants reflect on the influence of social interactions and daily activities on their experiences of fatigue. Through the combined emphasis on cognitive, behavioural, emotional, and psychosocial processes, the intervention operationalises a multimodal approach designed to foster comprehensive and sustainable behaviour change.

## Modifications

The timing of notifications sent through the app can be adjusted according to individual patient preferences. In addition, the duration of the intervention can be modified if necessary – for instance, in cases where patients or therapists are ill or on vacation. This flexibility allows for better alignment of the intervention with participants’ personal circumstances and enhances engagement with the programme.

## How well: Fidelity

Adherence and fidelity are continuously monitored through app-generated data and session reports. This enables real-time evaluation of participants’ engagement and the consistency of intervention delivery across sessions.

## Discussion

We present a novel blended care intervention for fatigue after brain injury, ‘Tied by Tiredness’. This intervention is a direct answer to the need for personalised and multimodal treatment approaches that acknowledge and incorporate the multifactorial nature of fatigue after brain injury.^22^

A strong point of this intervention is that the collection of micro-level data on patients’ lives accurately maps out, for each patient, which factors in daily life are associated with high (or low) levels of fatigue. This allows for highly personalised treatment approaches aimed at reducing fatigue symptoms.^23^ Furthermore, this intervention can improve self-management and may facilitate a sense of control by actively involving patients in their treatment process.^24^

Another strong point is the inclusion of weekly feedback sessions. These sessions are primarily designed to help both patient and therapist better understand fatigue and the factors that may contribute to it for this specific person. Each session focuses on a specific topic and builds on the previous one. Using insights gained from the experience sampling method app, the therapist offers personalised feedback, encouraging the patient to consider adjustments in daily behaviour that might help reduce fatigue symptoms. Regular feedback sessions based on experience sampling data, can help identify patterns and inform treatment adjustments, enhancing the therapeutic alliance.^25^ Intensive collaboration between patients and clinicians is recommended to ensure that experience sampling data is effectively tailored to the patient's unique circumstances, thereby enhancing its potential to improve clinical care.^25,26^

An added value of this intervention is its partly protocoled, partly person-oriented nature. The treatment protocol provides frameworks and possible starting points, but the actual content of the therapeutic interventions depends on the needs and context-specific factors of a patient.^27^ However, this may also be a limitation, because it calls on the patient and therapist to actively search for connections and provide targeted feedback and interventions. All of this costs time and energy, which may be seen by some therapists as an additional burden that can create a threshold for applying the intervention.^28^

Another potential barrier to using the intervention could be the lack of experience with mHealth, both on the part of the therapist and patient. A pre-existing feeling of being ‘not good with technology’ may lead to a lack of motivation in the therapist.^29^ The use of this intervention requires access to an experience sampling method app, as well as training in the intervention protocol and in interpreting the experience sampling data. For some healthcare institutions or therapists, this could present an obstacle to using the intervention.

Furthermore, in the pilot phase, the face-to-face intervention was compared with e-mail contacts but not with videoconferencing. In current rehabilitation practice, face-to-face sessions are the standard mode of treatment delivery, which guided the decision to apply this format in the present study. Nonetheless, videoconferencing represents a potentially valuable and less time-consuming alternative for both patients and therapists. Future research and implementation studies could explore the feasibility and effectiveness of delivering the intervention through videoconferencing to increase accessibility and flexibility of care.

An important limitation of this study is that the intervention may not be suitable for all patients with acquired brain injury and severe fatigue as measured with the Fatigue Severity Scale (score of 4 or higher). The intervention requires a certain level of cognitive and communicative abilities. Patients with severe cognitive impairments, such as reduced attention, memory problems, or diminished insight into their condition, may have more difficulty independently applying the offered strategies. Similarly, individuals with pronounced communication disorders could benefit less from interventions that rely heavily on verbal interaction and self-reflection. Other conditions, such as depression or somatic complaints known to influence fatigue, can also affect the intervention's effectiveness.

From a stepped care approach, this intervention may represent a first, low-cost step to understanding and managing fatigue for patients who are severely hindered by fatigue in their daily lives. Given the complex, multifactorial nature of fatigue after brain injury, persistent fatigue may require additional care in the form of more structured exercise training^30^ or person-centred therapy such as cognitive behavioural therapy. Although the Experience Sampling Method does not provide direct biophysiological measurements such as heart rate, it incorporates self-reported assessments of physical exertion. Experience Sampling Method studies can be combined with mobile health (mHealth) technologies and wearable sensors that do capture biophysiological data (e.g. heart rate monitors, accelerometers), thus allowing integration of self-reported experiences with objective physiological measurements. For example, m-Path Sense integrates the Experience Sampling Method with background mobile sensing from wearable sensors to link both self-reported and passively collected data. This approach enables event-contingent sampling protocols, such as triggering surveys upon entering predefined geofences. By combining active and passive data streams, this methodology enhances the precision of data collection and holds significant promise for digital phenotyping and the development of personalised interventions.^31^

The potential to extend the intervention with additional modules, for example, focusing on exercise or cognitive behavioural therapy, represents a valuable prospect for future research, particularly during the implementation phase.

The results of the Tied by Tiredness study on the costs and effectiveness of this intervention for fatigue after acquired brain injury, and the evaluation of the intervention process will be presented in ensuing papers. This intervention is the subject of an ongoing trial and preparatory work, in the form of a pilot study, has been done by the research team.^4^

## Supplemental Material

sj-docx-1-cre-10.1177_02692155251407318 - Supplemental material for Rationale and description of Tied by Tiredness: A blended care intervention for fatigue after acquired brain injury Supplemental material, sj-docx-1-cre-10.1177_02692155251407318 for Rationale and description of Tied by Tiredness: A blended care intervention for fatigue after acquired brain injury by Ela Lazeron-Savu, Tom Smejka, Bert Lenaert, Jeanette Dijkstra, Claire Wolfs, Vera Schepers, Rudolf Ponds and Caroline van Heugten in Clinical Rehabilitation

sj-jpg-2-cre-10.1177_02692155251407318 - Supplemental material for Rationale and description of Tied by Tiredness: A blended care intervention for fatigue after acquired brain injury Supplemental material, sj-jpg-2-cre-10.1177_02692155251407318 for Rationale and description of Tied by Tiredness: A blended care intervention for fatigue after acquired brain injury by Ela Lazeron-Savu, Tom Smejka, Bert Lenaert, Jeanette Dijkstra, Claire Wolfs, Vera Schepers, Rudolf Ponds and Caroline van Heugten in Clinical Rehabilitation

sj-jpg-3-cre-10.1177_02692155251407318 - Supplemental material for Rationale and description of Tied by Tiredness: A blended care intervention for fatigue after acquired brain injury Supplemental material, sj-jpg-3-cre-10.1177_02692155251407318 for Rationale and description of Tied by Tiredness: A blended care intervention for fatigue after acquired brain injury by Ela Lazeron-Savu, Tom Smejka, Bert Lenaert, Jeanette Dijkstra, Claire Wolfs, Vera Schepers, Rudolf Ponds and Caroline van Heugten in Clinical Rehabilitation
