# Supplementary material for: Rationale and description of Tied by Tiredness: A blended care intervention for fatigue after acquired brain injury
Source: Clin Rehabil. 2025 Dec 16;40(5):575–86. doi: 10.1177/02692155251407318 (PMC13121811; doi:10.1177/02692155251407318)
Supplement: sj-docx-1-cre-10.1177_02692155251407318 - Supplemental material for Rationale and description of Tied by Tiredness: A blended care intervention for fatigue after acquired brain injury [file sj-docx-1-cre-10.1177_02692155251407318.docx]

**Rationale and description of Tied by Tiredness: a blended care intervention for fatigue after acquired brain injury**

**Supplementary material**

**Illustration of a case description, case John.**

This supplementary material provides a detailed account of the progression and content of the sessions conducted during the Tied by Tiredness, a 6-week blended-care intervention.

Each section summarizes the key activities and adjustments made throughout the process, offering additional insights to support the main case report.

**Case introduction and types of fatigue**

It concerns a 61-year-old man named John who suffered a stroke (two years prior to following the Tied by Tiredness intervention) and dissection of the right internal carotid artery. He is married, has three adult children, and works full-time as a social psychiatric nurse. John followed a rehabilitation program immediately after the stroke with an emphasis on physical recovery, but his fatigue remained being seriously disabling both at home and at work.

At the start of Tied by Tiredness intervention, John reported a score of 7 on the Fatigue Severity Scale (range 1-7). During the intervention, the average response rate of John to the experience sampling measurements was 75.6%.

***Types of fatigue, session 1*** - Because his fatigue has been present for over two years, John has read a lot of information about it. The psycho-educational part of the intervention has been discussed briefly. John indicates that he knows how it works, the theory, but that he is unable to change his fatigue. This causes a lot of frustration. Figure 1 and 2 shows a graphical timeline of the course of fatigue during the first week. John indicated that he usually woke up refreshed in the morning, but during the day, fatigue increased rapidly and in the evening it was highest. He noticed that there is a large variation in fatigue and that there are moments when he feels less tired. The wide variation in fatigue was discussed and it was considered what small adjustments can already be made, to reduce the peaks. John indicated that he would try to take a rest break in the morning and afternoon. In the graphical timeline representation of fatigue, John saw that there was a big difference between physical and mental fatigue. Mental fatigue contributes the most to the feeling of being tired. John indicated that he experiences mental fatigue in particular, but he did not realize that mental fatigue was so strong.

[insert supplementary material Figure 1]

[insert supplementary material Figure 2]

**Fatigue and feelings in relation to fatigue- session 2**

This week, attention was paid to various feelings in relation to fatigue. Feelings of sadness, anxiety or feeling stressed were rarely reported by John. Feelings of helplessness and feeling insecure were more often indicated, but there was no significant correlation with fatigue. In previous psychological treatment, John has information about the principles of cognitive behavioural therapy. The information he received also helps him, to a certain extent, in dealing with the fatigue complaints and their consequences. Less helpful thoughts about pushing boundaries did come forward. Guilty thoughts and thoughts that he has little influence on fatigue also came forward. The optional information about how to deal with unhelpful thoughts was discussed with John, as he indicated that he would like a refresher.

With regard to the course of fatigue, there were still peaks, but there were fewer moments when John gave a 7 as a score for fatigue. John indicated that he takes more breaks, this is not always possible, partly due to the unhelpful thoughts about pushing boundaries.

**Fatigue in relation to mental and physical effort – session 3**

John indicated that he had thought about the previous weeks. He thought he had no obstructive thoughts about fatigue, but when filling in the questions in the app, unhelpful thoughts still came up, as discussed in the previous conversation. He has reviewed the information about unhelpful thoughts and has come to realize that thoughts of guilt influence the tendency to push his boundaries and take few breaks. The theme of this week, the degree of physical exertion in relation to fatigue, but also the way of taking breaks were discussed. An explanation was given about what taking breaks means for mental rest, namely in a low-stimulus environment (no mobile phone, TV or worrying) and for physical rest (relaxation for the muscles).

Looking at the weeks before, it became obvious that John was often less physically tired. High scores for mental and general fatigue were common. There was a high positive correlation between mental effort and mental fatigue. John indicated that he exerts himself most mentally at work and also that he is unable to take breaks because he shares his office with a colleague. The information from the graphs and the high positive correlations hit John hard. He had not realized that the level of mental effort had such a big impact on his fatigue. John indicated that he would look at his work to see what changes could be made so that he could still take breaks.

***Fatigue in relation with activities – session 5***

The relationship between activities performed and the degree of fatigue was discussed this week. John has spoken with his colleagues, and he is now given the opportunity to use a room alone when he wants to rest. When looking at the activities performed and their relationship with fatigue, "working" and "talking, in conversation" are most prominent. Also, during other activities such as "resting" or "watching TV" the fatigue scores are high, especially mental fatigue. The course of fatigue in recent weeks showed fewer peaks, most high scores were around score five. John gave these elevated scores when he was talking to others.

**Fatigue *in relation with* social contact and evaluation of treatment – session 6**

This week the relationship between fatigue and location was examined. As expected, being at work, John experienced the most fatigue, mental fatigue. When looking at the relationship between fatigue and who he was with, John experienced the most fatigue in the company of friends and/or family. John indicated that he had not thought about this before. By discussing this, John indicated that he feels a kind of pressure to be at his best with friends and family and that he may then use extra energy. He wants to show that he is doing well and therefore he unconsciously goes beyond his limits. John indicated that he was happy with these insights, and he was going to try to pay attention to this and be less active in these kinds of companies.

The course of fatigue in recent weeks, during the intervention, was examined, see figure 2. It is noticeable that since week three there were no more scores of seven and that since week five there were fewer fluctuations in the scores.

Response from John, during evaluation of the intervention "First of all, it was nice to have a follow-up to be held up to a mirror again. Knowing is different from doing. I don't feel like I'm alone in this. As a result of the intervention, I adjusted my rest times afterwards. I didn't rest at fixed times, which resulted in high peaks. I wasn't strict enough in this and now that the rest time is central, I notice that my energy management has improved. If things go better one day, this doesn't mean that the next day will be the same."
