# Supplementary figures and images for: Rationale and description of Tied by Tiredness: A blended care intervention for fatigue after acquired brain injury
Source: Clin Rehabil. 2025 Dec 16;40(5):575–86. doi: 10.1177/02692155251407318 (PMC13121811; doi:10.1177/02692155251407318)

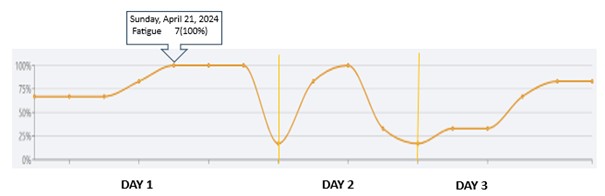

Supplement: sj-jpg-2-cre-10.1177_02692155251407318 - Supplemental material for Rationale and description of Tied by Tiredness: A blended care intervention for fatigue after acquired brain injury [file sj-jpg-2-cre-10.1177_02692155251407318.jpg]

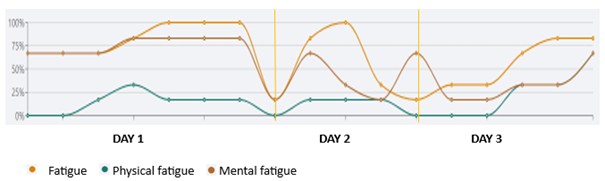

Supplement: sj-jpg-3-cre-10.1177_02692155251407318 - Supplemental material for Rationale and description of Tied by Tiredness: A blended care intervention for fatigue after acquired brain injury [file sj-jpg-3-cre-10.1177_02692155251407318.jpg]
